# Improved parameter fitting for models of young and aged neurons

**DOI:** 10.1186/1471-2202-12-S1-P207

**Published:** 2011-07-18

**Authors:** Christina M Weaver, Aniruddha Yadav, Patrick R Hof, Jennifer I Luebke

**Affiliations:** 1 Department of Mathematics, Franklin and Marshall College, Lancaster, PA 17603, USA; 2 Department of Neuroscience and Friedman Brain Institute, Mount Sinai School of Medicine, New York, NY 10029, USA; 3 Department of Anatomy and Neurobiology, Boston University School of Medicine, Boston, MA 02118, USA

## 

The parameters of neuronal compartment models must be determined carefully in order to match experimental data. Optimization algorithms can simplify this task by automatically searching the multidimensional parameter space to identify combinations of parameters that best fit experimental data, as measured by a fitness function that represents salient differences between simulated and experimental data. The success of automated parameter fitting depends critically on many issues, including the choice of parameters selected for fitting, the parameter search method, and the design of the fitness function. Previously we developed a fitness function that explicitly quantifies the shape of action potentials and afterhyperpolarizations [1], and the time-varying firing rate [2].

The present project arises from our study of neocortical pyramidal cells from young and aged rhesus monkeys in vitro [3]. In response to current clamp protocols that evoked action potentials, aged neurons fired at significantly higher rates than young ones did. Yet, both young and aged neurons displayed an initial fast phase of firing rate adaptation followed by a slower one. Neither parameter optimization with our existing fitness function, nor the popular phase-plane method [4], sufficiently captured the two-phase adaptation.

We have extended our fitness function to fit firing rate time series with two single exponentials, separated by an automatically-chosen cutoff. The fitness function also includes a term to quantify action potential backpropagation, whereby voltage decays approximately exponentially with distance from the soma [5]. The fitness function is designed for use with NEURON, with the parameter search guided by MATLAB’s Global Optimization Toolbox. We used the fitness function and search method to optimize morphologically accurate compartmental models of young and aged neurons to their respective physiological data. We compared results from our original and improved fitness functions against the phase-plane fitness function, separately optimizing either ion channel densities or channel kinetics. All searches identified multiple combinations of parameters that fit the data, with our new fitness function having the best qualitative matches to empirical data. These parameter fits will be used to identify biological mechanisms that may be responsible for the physiological differences between young and aged neurons.
